# A Systolic Array-Based FPGA Parallel Architecture for the BLAST Algorithm

**DOI:** 10.5402/2012/195658

**Published:** 2012-09-04

**Authors:** Xinyu Guo, Hong Wang, Vijay Devabhaktuni

**Affiliations:** ^1^Electrical Engineering and Computer Science Department, The University of Toledo, MS.308, 2801 W. Bancroft Street, Toledo, OH 43607, USA; ^2^Department of Engineering Technology, The University of Toledo, MS.402, 2801 W. Bancroft Street, Toledo, OH 43606, USA

## Abstract

A design of systolic array-based Field Programmable Gate Array (FPGA) parallel architecture for Basic Local Alignment Search Tool (BLAST) Algorithm is proposed. BLAST is a heuristic biological sequence alignment algorithm which has been used by bioinformatics experts. In contrast to other designs that detect at most one hit in one-clock-cycle, our design applies a Multiple Hits Detection Module which is a pipelining systolic array to search multiple hits in a single-clock-cycle. Further, we designed a Hits Combination Block which combines overlapping hits from systolic array into one hit. These implementations completed the first and second step of BLAST architecture and achieved significant speedup comparing with previously published architectures.

## 1. Introduction

In the bioinformatics and computational biology (BCB) domain, biological sequence alignment is a quite common task. It includes comparison of DNA (nucleotide), RNA (nucleotide), and protein (amino acid) sequences. In such comparison, a query sequence is aligned to subject sequences from a large database to find similarities [1]. Scientists are used to adopting sequence alignment to infer biological information of a newly discovered sequence from a set of previously known sequences. For instance, if a recently discovered sequence is similar to a known disease gene, then the biological information about the functionality of the new sequence can be inferred. This is extremely meaningful in early disease diagnosis and drug engineering [2]. In addition, biological sequence alignment plays an important role in the study of evolutionary development and the history of species [3]. 

**Figure 1:**
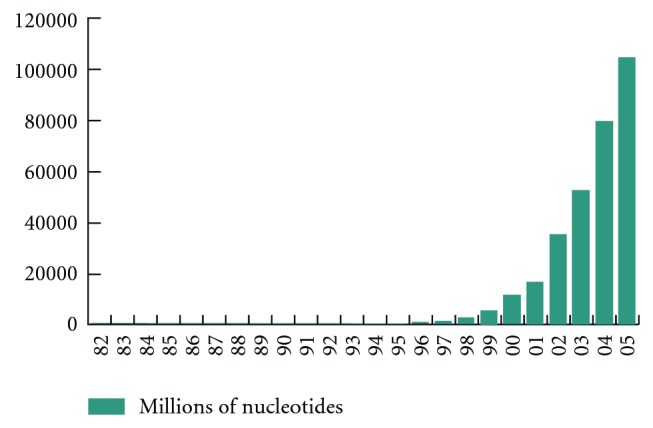
Exponential growth of biological sequence database on a yearly basis.

Being one of the most important tools applied in finding biological sequence alignment, BLAST (Basic Local Alignment Search Tool) [4] has been widely implemented on commodity PC clusters. But with the exponential growth of the biosequence databases (see Figure 1 [1]), meeting the computational requirements using current platforms is becoming a difficult task. 

Compared to general-purpose PC microprocessors, Field Programmable Gate Arrays (FPGAs) are able to provide a much higher degree of bit-level data parallelism which leads to higher computational speed. In addition, FPGAs are re-programmable [5]. Scientists have been investigating parallel architecture design for the BLAST algorithm on FPGAs to achieve higher computational efficiency. 

This paper proposes a design and implementation of a parallel architecture on FPGA for BLAST, namely, systolic array-based parallel architecture for the BLAST algorithm. The design is implemented with Very High Speed Integrated Circuit Hardware Description Language (VHDL), making it compatible across a number of FPGA architectures (e.g., Xilinx, Altera). The rest of this paper is organized as follows. We first present the overview of the BLAST algorithm as well as the related work. Then, the design of our FPGA architecture will be detailed. After that, architecture performance including storage resource requirements, FPGA resource consumption, and estimated speedups against other architectures will be analyzed based on synthesizing, functional, and timing simulation. Finally, conclusions and future work are presented.

## 2. The BLAST Algorithm

Biological sequence alignment algorithms can be exhaustive, meaning they give optimal alignments. Two examples are the Needleman-Wunsch [6] and Smith-Waterman [7] algorithms. Algorithms like BLAST are heuristic and give sub-optimal results [8]. Although heuristic algorithms produce local alignments that are not always optimal, they are normally much quicker than exhaustive dynamic programming algorithms. Exhaustive algorithms typically have a complexity of *O*(*mn*) [9]. The execution time of biological sequence alignment is crucial to bioinformatics scientists, especially due to the fact that the size of the biosequence databases has grown exponentially over years. BLAST is a very popular heuristic algorithm that provides high computational speed. 

The three main steps of the BLAST algorithm are finding hits, ungapped extension, and gapped extension. These are described as follows.

**(1) Finding Hits**

In this step, we use a protein sequence which consists of eight residues (amino acids) as an example shown below:

 PQGEFGVY.

The query sequence is divided into overlapping *k*-words as illustrated below with *k* = 3:

 Word 0: PQG
 Word 1: QGE
 Word 2: GEF
 Word 3: EFG
 Word 4: FGV
 Word 5: GVY.

Six words are extracted from this sequence. Generally speaking, (*m* − *k*) + 1 words would be extracted when the size of the sequence is *m*. After dividing the query sequence, these *k-words* plus a score matrix are used to find the possible matching words which have a score with at least the threshold value of *T*. Finally, subject sequences in the database are scanned one by one to find exact matches to those possible matching words. Previous work shows that this step could be finished by high-level application software running on a PC [10]. By designing an architecture which can implement every step of BLAST on a FPGA, the interface issue for software and hardware connection can be avoided, saving execution time. Instead of finding exact matches to those possible matching words, in this paper, the implementation searches for the exact matches of those *k*-words directly on the FPGA. This is called “finding hits.”

**(2) Ungapped Extension**

In this step, each hit recorded in the last step is extended by aligning with a subject sequence in both directions without any gap. A score matrix, for example, BLOSUM50 [10], is used to compute residue pair alignment scores. The extension process is terminated when the total score of the alignment is below a threshold value. These results from ungapped extension are called high-scoring segment pairs (HSPs).

**(3) Gapped Extension**

In this stage, the Smith-Waterman or Needleman-Wunsh algorithm is applied to perform the gapped alignment in both directions for those collected HSPs. After this step, a collection of homologous sequences are assigned with different scores.

We choose the Needleman-Wunsch algorithm for gapped extension implementation because of its ability to find optimal global gapped alignment between the query sequence and the subject sequence [8]. Suppose that there are two sequences, *Q* and *S*, whose lengths are *M* and *N*, respectively. *Q*[1 : *i*] denotes the first *i* characters in sequence *Q* and *S*[1 : *j*] denotes the first *j* characters in sequence *S*. The similarity score between *Q*[1 : *i*] and *S*[1 : *j*] can be obtained from the similarity scores computed for *Q*[1 : *i* − 1] and *S*[1 : *j* − 1]. A dynamic programming score matrix *D* is built, where each element of the matrix denotes the best alignment score between the *Q*[1 : *i*] segment of *Q* and the *S*[1 : *j*] segment of *S*. 

The first step of Needleman-Wunsch algorithm is to create the score matrix *D* with *M* + 1 columns and *N* + 1 rows. *M* and *N* are lengths of sequences *Q* and *S*. The first row and the first column are assigned the value zero. The computation complexity of the initialization step is *O*(*M* + *N*). 

The second step is comprised of filling the matrix. D_i,j_ is used to express the *cell *(*i*, *j*) of the scoring matrix *D*. *D*_*i*,*j*_ is defined as the maximum of the similarity scores computed in positions (*i* − 1, *j* − 1), (*j*, *j* − 1), and (*i* − 1, *j*) associated with gap penalty or *S*_*i*,*j*_. The following equation is used to compute the value of the* cell *(*i*, *j*):

(1) D(i,j)=max⁡{D(i−1,j−1)+Si,j,D(i−1,i)+d,D(i,j−1)+d.

In this equation, *S*_*i*,*j*_ is the scoring matrix score for X_i_ and *Y*_*j*_. *S*_*i*,*j*_ = 1 if the *i*th character in *Q* is equal to the *j*th character in *S* (MATCH score); otherwise S_i,j_ = 0 (MISMATCH penalty). *d* is the gap penalty, *d* = 0. *D*(*i* − 1, *j*) + 1 is the score of an alignment between *X*_*i*_ and a gap in *Y*. *D*(*i*, *j* − 1) + *d* is the score of an alignment between a gap in *X* and *Y*_*j*_.

**Figure 2:**
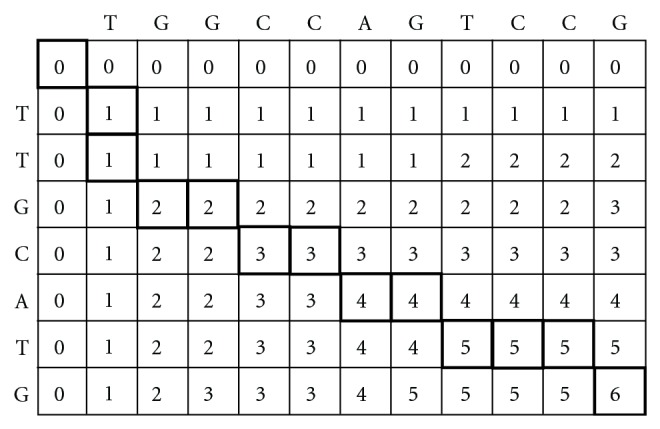
Illustration of Needleman-Wunch algorithm.

The last step of this algorithm is trace back. Trace back can form the best global alignment between *Q* and *S*. It starts from the bottom-right value in the matrix and ends at the up-left value. For each step, the next alignment pair which is added to the front of the alignment segment accords to 3 rules: (1) If the *cell *(*i*, *j*) was derived from *cell *(*i* − 1, *j* − 1), the pair of alignment *X*_*i*_ and *Y*_*j*_ is added to the front of the alignment segment. (2) If *cell *(*i*, *j*) was derived from *cell *(*i* − 1, *j*), *X*_*i*_ and a gap in *Y* are added to the front of the alignment segment. (3) If *cell *(*i*, *j*) was derived from *cell *(*i*, *j* − 1), a gap in *X* and *Y*_*j*_ are added to the front of the alignment segment. In some cases, the same similarity scores exist among those three input cells. It's hard to decide which cell *cell *(*i*, *j*) comes from. The diagonal cell is chosen under this situation to shorten the length from the bottom-right cell to the upper-left cell. The shortest alignment segment presents the best global alignment between two sequences.  Figure 2 shows an example which illustrates the Needleman-Wunch steps. It corresponds to the following alignment: T_G G C C A G T C C G and T T G_C_A_T_G.

## 3. Previous Work 

FPGAs not only provide high speedups and high bit-level data parallelism compared to regular processors, but also include the convenience of reprogrammability. More and more bioinformatics scientists have designed parallel FPGA architectures for the BLAST algorithm in order to solve the biological sequence alignment issue [11]. Many architectures have been proposed already such as Mercury BLASTn [12–14], Tree-BLAST [9], Mercury BLASTp [15], RC-BLAST [16], FPGA/FLASH Accelerator [17], Multiengine BLASTn Accelerator [18, 19], and many industry applied systems like BEE2 [20] and Mitrion [21]. Most of them adopt the Word Position Record-Based Search (WPRBS) method, which constructs all storage tables to record the position of each word in query sequence, then sends those words to the accelerator one by one where the storage table is searched to find the hit. Although this approach is widely used, it still suffers from time and storage limitations.

Using WPRBS, at most one hit can be found in one-clock cycle because only one word can be searched per clock cycle. FPGA memory port number also limits WPRBS searching capacity no matter where the storage tables are stored: internal chip memory or external chip memory. Take storing tables in an external memory for instance, Mercury BLASTn [12] and Mitrion [21] built a hash table from the query. During the searching process, an accelerator checks words in the subject sequence database against the hash table one by one. An external SRAM attached to the FPGA is used to store the hash table since the internal block RAMs do not have enough storage space to hold tables for long query sequences. This situation obviously causes a timing issue: accessing delay to external SRAM leads to long pipeline cycle time. FPGA/FLASH [17] could minimize the timing issue above. The database is formatted as an index structure in which every word is associated with its position in the sequence and its adjacent environment so that short, ungapped alignments could be computed simultaneously, avoiding many random accesses to the database. But the size of the database index is very large, A 150 GB index for the human genome is needed when storing a 40 amino acid substring environment. This requires 50 times more storage than the raw data [22]. In the future, with the exponential growth of the database, the storage resource requirement will become a serious issue. To get better searching efficiency, Multiengines BLASTn [18] adopted 64 identical computing units in a single chip to compare the query with 64 subject sequence in database at the same time. Mercury BLASTp [15] proposes a two-hit generator for accelerating the first stage of BLASTp. Both of these two methods are spawned from WPRBS. 

 Another searching strategy is based on the systolic array without building any table. Systolic array has been implemented for exhaustive algorithms like Needleman-Wunsch [10]. Implementing BLAST using systolic array is a relatively newer research area [9]. 

## 4. Parallel Architecture Design of BLAST Algorithm with Multiple-Hits Detection

**Figure 3:**
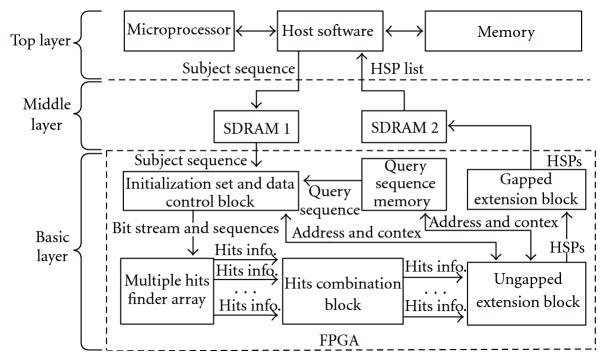
The structure of the project.

As Figure 3 shows, the proposed BLAST searching system contains three layers. The top layer is a host computer which runs high-level application software. The middle layer consists of two SDRAMs—one is used as a subject sequence database and the other is used as a results container which receives HSPs lists from the basic layer. The basic layer is the systolic array-based parallel architecture fitted on FPGA. This layer scans all subject sequences from database for the query sequence in order to get a HSPs list. After that, the list is sent to the host computer software that assigns statistical significance specified by bioinformatics scientists.

In the top layer of this system, a Dell Precision T1500 desktop computer with an Intel Core i7 microprocessor and 4 GB memory runs the host software. In the middle layer, two 1 GB SDRAMs are attached to the FPGA where the basic layer is located. The basic layer is the systolic array-based parallel architecture that implements the BLAST algorithm. This layer is mapped to one FPGA chip (Xilinx Virtex-5 XC5VLX110T) which is embedded on the Xilinx ML509 Evaluation Platform. It contains 6 different blocks with various functions. The Initialization Set and Data Control Block is used to initialize the status of registers for the Hit Finder Array and to forward subject sequence as well as query sequence to the Hit Finder Array. The Query Sequence Memory storing query sequence is constructed from internal block RAMs. The four other blocks implement 3 steps of the BLAST algorithm in parallel. Hits Finder Arrays can detect multiple hits per clock cycle. The Hits Combination Blocks can find combinable overlapping hits and combine them. For example, it can find adjoined overlapping hits “TKEL” and “KELP” then combine them into “TKELP”. Ungapped Extension Blocks extends ungapped hits in both directions until the score meets a thread value *T*. Gapped Extension Block extension gapped extension on HSPs through exhaustive algorithm like Needleman-Wunsch. The block outputs HSPs and alignments scores.

### 4.1. Multiple Hits Detection Module 

**Figure 4:**
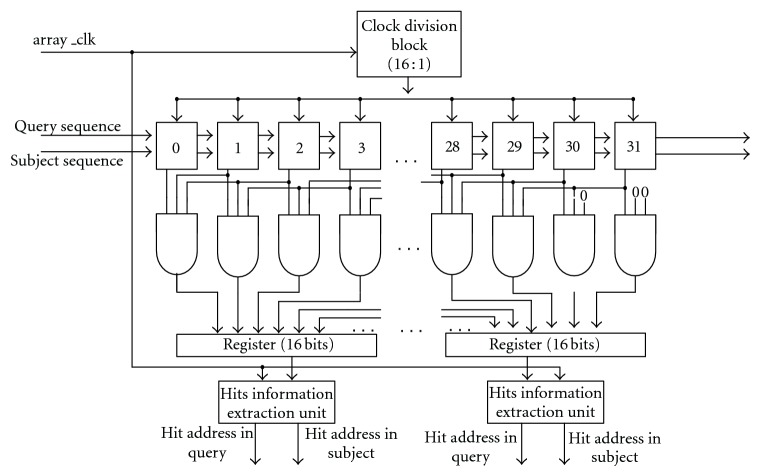
Architecture of multiple hits detection module.

Multiple Hits Detection Module is used to detect 3-word hits and record the hits' address in the query and the subject sequence. Compared to WPRBS method which could detect at most one hit in one clock cycle, this design can detect multiple hits in only one clock cycle. The architecture of Multiple Hits Detection Module is shown in Figure 4.

**Figure 5:**
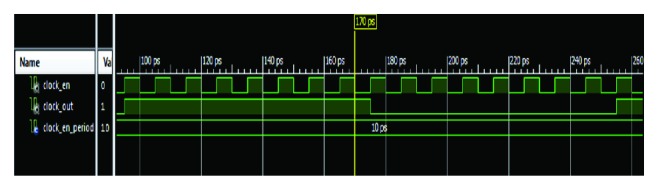
Behavior simulation of clock division block.

As the figure illustrated, there is a systolic array with 32 processing units, every 3 units are connected to one 3-input AND gates. Every 16 gates' outputs are connected to a 16-bit register. The value of each register is sent to the corresponding Hits Information Extraction Units for recording the hits address in the query and the subject sequence. The systolic array and the Hits Information Extraction Unit are driven by two different clocks. Clock Division Block is designed to generate necessary clocks. The behavior simulation of Clock Division Block is shown in Figure 5.

clock_en (array_clk in Figure 4) drives Hits Information Extraction Units; clock_out is the clock source of the systolic array. These two clocks work together driving a pipelined architecture without any pause. At each clock_out rising edge, query or subject sequence moves forward for one processing unit; at each clock_en rising edge, a bit value at a certain position in each register is used to detect the location of hits.

**Figure 6:**
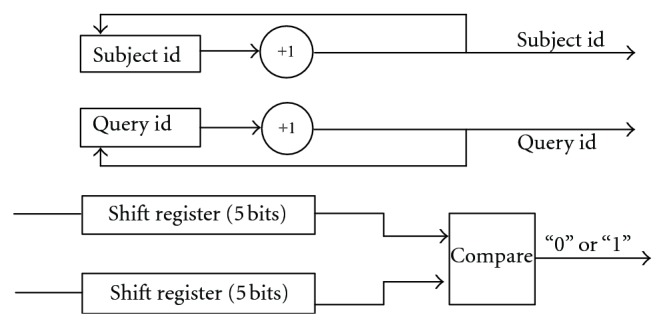
Architecture of processing unit of multiple hits detection module.

The whole architecture works as follows: first, a query sequence with 32 characters is forwarded into the systolic array so that each processing unit holds a character from the query sequence. Then the subject sequence is driven in to the systolic array by each internal clock rising edge. Meanwhile, the incoming subject character and the query character which are held by the unit are compared, if they are identity, the logic “1” would be generated; otherwise, the logic “0” would be generated. The comparison result is an input of a 3-input AND gate. Every 3 processing units maps to an AND gate. A hit is detected when the logic “1” is generated from its output. So, the systolic array with 3-input AND gates can detect multiple hits at one internal clock rising edge. The architecture of the processing unit in the systolic array is illustrated in Figure 6.

In the implementation, 20 3-bit long binary numbers (“00001” to “10100”) are defined to present 20 amino acids in a protein. Shift registers are able to shift characters to the adjacent processing unit so that the query and the subject sequence can go through the systolic array. When a new character from the query or the subject sequence is shifted into the unit, the corresponding id register would increase its value by 1. Each processing unit of the systolic array will compare two characters in it at each internal clock rising edge. 

As shown in Figure 4, outputs of 32 3-input AND gates goes into 2 16-bit registers. The Hits Information Extraction Unit detects hits' locations and record them. The algorithm running in this module is presented in Pseudocode 1.

The Multiple Hits Detection Module is a parallel, pipelined architecture. The systolic array with 32 processing units cooperates with 32 3-inputs AND gates to detect hits in both sequences. Hits Information Extraction Block records those hits' locations. 

### 4.2. Hits Combination Block

If there is high similarity between query and subject sequence, the Multiple Hits Detection Module may output a large amount of hits per clock cycle. Ungapped Extension Block is relatively slower than Multiple Hits Detection Module and the systolic array in this situation. As a result, the processing speed of these two stages is unbalanced. 

Previous research [23, 24] suggested that in many cases there is a high similarity between the query sequence and the subject sequence. Many overlapping hits can be reported by adjacent components at the same time. Processing those hits not only wastes the execution cycle for Multiple Hits Detection Module, but also consumes additional extension time for Ungapped Extension Block because one hit is extended twice. For instance, two adjacent hits “ATK” and “TKP” are found but they are actually one hit “ATKP”. If they are not combined into one hit, they would have been recorded twice in Multiple Hits Detection Module and extended twice in Ungapped Extension Block. In this section, we implement Hits Combination Block to address this issue. It can detect the overlapping hits and merge them to reduce verbose hits and maintain the sensitivity of BLAST. 

**Figure 7:**
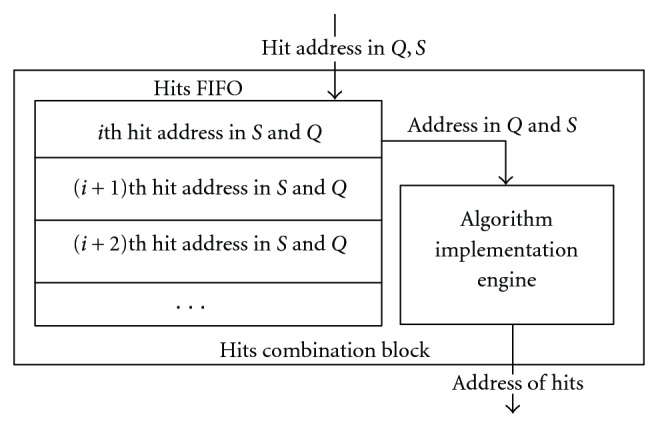
Hits combination block.

This block contains a Hits (First In First Out) FIFO buffer which is used to store hits location addresses from both query and subject sequence. The algorithm implementation engine executes hits combination algorithm then forward the hit addresses and hit length to the Ungapped Extension Block. The data flow in this block is shown in Figure 7.

The order of the Hit addresses in the Hits FIFO buffer follows the direction of the stream flow (Figure 5). For example, the address of “AKL” should be stored in the FIFO buffers first, then the “KLP”. The combination algorithm is triggered when there is more than one record in the Hits FIFO buffer. Here, the hit addresses in subject sequence are recorded and forwarded to the following operations. First, the algorithm implementation engine calculates the ending address of the *i*th and (*i* + 1)th hit in subject sequence. In this paper, the starting address is the recorded address minus 1, because the recorded address is the address of the middle word in a 3-word hit. The ending address is the address of the last character in the sequence, so (Ending  address = Starting  address + (seqence  length − 1)). Here, the sequence length is equal to 3. Then, a comparison between *i*th hit's ending address and (*i* + 1)th hit's starting address is performed. If ith hit's ending address is greater than or equal to the (*i* + 1)th hit's starting address, there would be an overlap between two hits. The algorithm can determine new addresses in both subject sequence and query sequence of the merged hit and calculate its length. In the next step, the algorithm records the new address and hit length at the ith place in Hit's FIFO buffer. If *i*th hit's ending address is less than the (*i* + 1)th hit's starting address, the (i + 1)th hit address will be chosen to be compared with (*i* + 1)th hit address. This operation continues until the Hits FIFO buffer is empty.

### 4.3. Parallel Architecture for Multiple Hits Detection Module and Hits Combination Block

**Figure 8:**
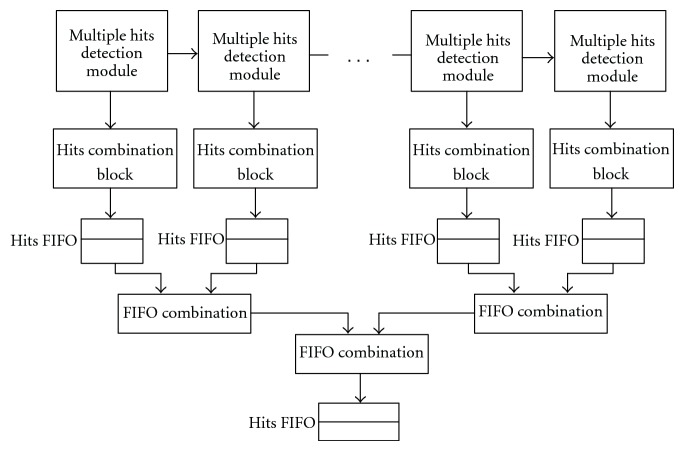
Parallel architecture of multiple hits finder array and hits combination block.

Because Multiple Hits Detection Module can detect multiple hits in one internal clock cycle, multiple modules working together in parallel speeds-up hit detection. In order to construct a longer array, many Multiple Hits Detection Modules are connected serially.  Figure 8 shows the way of the connection.

As Figure 8 shows, the large systolic array contains many Multiple Hits Detection Modules. Every module matches one Hits Combination Block, each of which records and combines hits detected by its mapped module. After that, it sends the hits information to a local hits FIFO. Then FIFO Combination modules combine hits FIFOs and deliver hits information to larger hits FIFOs. In this architecture, Multiple Hits Detection Modules are connected in a serial way to extend the array comparison size. Each Hits Combination Block maps to a Multiple Hits Detection Module so that each block can detect overlapping hits and merge them simultaneously. The parallel implementation cost more on-chip resource to trade for speed. 

### 4.4. Ungapped Extension Block

**Figure 9:**
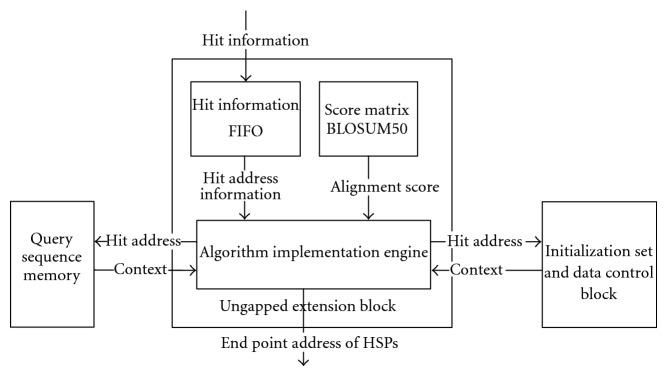
Ungapped extension block.

**Figure 10:**
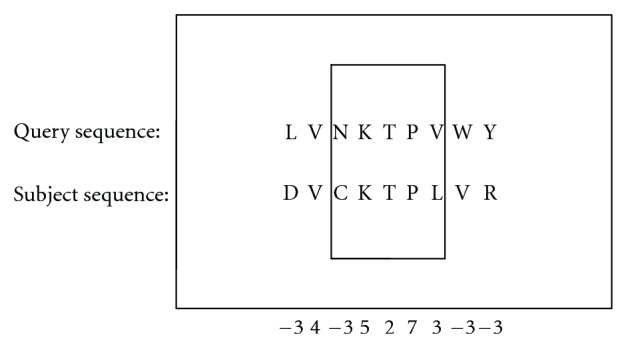
Extension procedure.

This block implements the ungapped extension step of the BLAST algorithm. FIFO is used to store the hits information. The score matrix BLOSUM50 [10, 25] is applied to find the alignment score for a pair of words. If this block detects a hit in a FIFO, the address of the hit word in query sequence, and subject sequence and the hit length would be extracted from the FIFO to compute both extension points. After this step, the Ungapped Extension Algorithm Implementation Engine can finish the ungapped extension step. The data flow in the Ungapped Extension Block is shown in Figure 9. The extension procedure in Algorithm Implementation Engine is illustrated in Figure 10.

The following is the procedure to find the hit KTP. After computing the addresses of characters “K” and “P,” the starting addresses of the extension, the algorithm drives the extension in both directions. Each pair of the residues own one alignment score which can be found in the BLOSUM50 score matrix. In Figure 13, the alignment score “L” and “D” is −3, and the score of the pair “V” and “V” is 4. During this extension procedure, the total score of these alignment pairs is computed. Ungapped extension step stops when the total score is more than a threshold. Then endpoints of this high-scoring segment in both directions on both query and subject sequences are recorded and forwarded to the Gapped Extension Block for gapped alignment.

**Figure 11:**
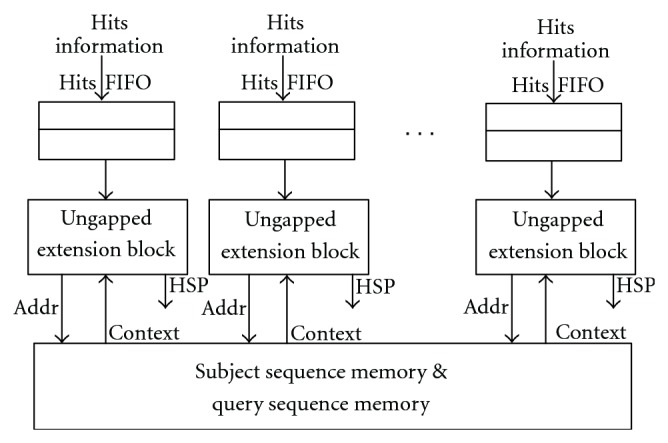
Parallel architecture for ungapped extension block.

In this step, hits which are sent from Hits FIFO of the Hits Combination Block are extended to either side to identify a HSP. The extension process needs to be triggered by at least one hit detection. Mercury BLASTn prefilters the hit's characters because only one hit can be searched at one clock cycle. FPGA/FLASH could get the subsequence directly because it constructs the index for each word. Compared to methods applied in Mercury BLASTn and FPGA/FLASH, Multiple Hits Detection Module is more efficient since it could detect multiple hits in one clock cycle. However, the serial ungapped extension process is slower than Multiple Hits Detection Module. To speedup this step, we proposed two methods in the above sections: adding Hits Combination Block between Multiple Hits Detection Module and Ungapped Extension Block; dividing the long components array into several parallel components array groups. As shown in Figure 11, we propose one more parallel architecture for Ungapped Extension Block.

In this architecture, each Hits FIFO of Hits Combination Block connects to an Ungapped Extension Block. Each block connects to the query sequence memory as well as Initialization Set & Data Control Block because the context of the hits is necessary for extension. System may slow down if hits from all Hits FIFOs are queued in only one Ungapped Extension Block. In this architecture, hits information could be distributed into different paths and save processing time.

### 4.5. Gapped Extension Block

**Figure 12:**
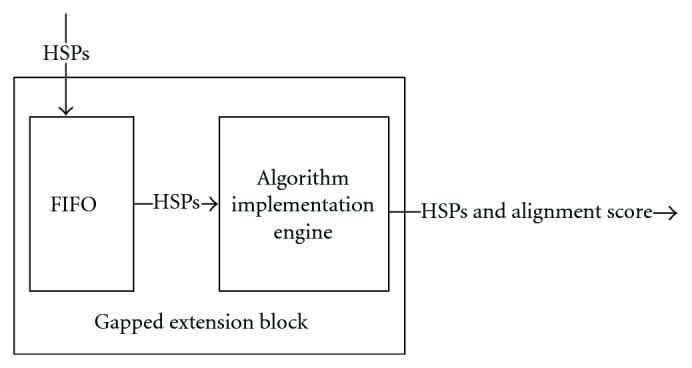
Gapped extension block.

In this block, HSPs from the FIFO buffer are used to perform gapped extension. The Gapped Algorithm Implementation Engine will be triggered once the block detects that there is a pair of segments in FIFO. We propose using the Needleman-Wunsch algorithm to finish the gapped extension.  Figure 12 shows the dataflow in Gapped extension block.

This paper focuses on the first two steps of BLAST algorithm including Finding Hits and Ungapped Extension because they consume most execution time in BLAST algorithm. In order to implement the Needleman-Wunsch algorithm on the FPGA, some efficient architectures [26, 27] are already designed. Efficient implementation should be easily done based on these previous research.

## 5. Performance Analysis 

We have mapped Multiple Hits Detection Module, Hits Combination Block, and Ungapped Extension Block architecture on the Xilinx XC5VLX110T FPGA which is embedded on ML509 education board. It is also referred as XUPV5-LX110T board. The architecture was designed under Xilinx ISE Design Suite 12.1 in Very High-Speed Integrated Circuit Hardware Description Language (VHDL). 

In our system, the Xilinx ML509 education board has a PCI Express(X1) interface which could provide 500 MB/s throughput. The board is then connected to the host computer: Dell Precision T1500 PC with a 2.80 GHz Intel Core i7 microprocessor and 4 GB memory. NCBI BLAST software runs on the host. In our experiment, a systolic array-based parallel architecture with 2048 processing units was implemented on the Xilinx ML509. Except for this implementation, we also mapped systolic array based architectures with 1204, 2048, and 3072 processing units to different FPGA devices under Xilinx ISE Design Suite. All performance data came from the design report. The clock values came from the timing analyze tool called postplace and route which integrated in the ISE. We will analyze the architecture performance from two aspects: one is comparing our architecture with previous architectures on hardware, the other is comparing our architecture against BLAST software versions. For hardware comparison, we focus on 3 parts including synthesis performance, storage requirement, and word scanning speed. For software versions comparison, we focus on the execution time analysis. 

### 5.1. Hardware Performance Comparison

 In this section, a hardware performance comparison is made between ours and other FPGA architectures from 3 aspects including synthesis performance, storage requirement, and word scanning speed. Tree-BLAST and Mercury BLASTn are WPRBS architectures, families of FPGA-based accelerators is a systolic array-based architecture. Advantages of our architecture would be highlighted through the comparison. All data which reflects the capability of architectures in the comparison from previous publication. 

#### 5.1.1. Synthesis Performance

Tree-Blast adopts systolic array architecture. Because every four components need a BRAM to index the scoring matrix, the input array size the system could process is restricted by BRAM. As a result, the query size is limited up to 600 on XC2VP70 and 1024 on XC4VLX160. Xia et al. [28] implemented a FPGA-based accelerator which is also a systolic array-based one for BLAST algorithm called families of FPGA-based accelerators. The processing unit in their architecture is more complicated than ours. Their processing unit needs more registers to get match information from two adjacent units. In addition, the data stream in the array needs to be shut down to record hits addresses. As a result, it needs more registers to store the systolic array status in order to resume it. In our architecture, WPRBS is replaced by Multiple Hits Detection Module to find hits so that the BRAM is not the bottleneck of array size anymore but LUTs are. We can build 2048 components on XC5VLX110T which is two times the array size of Tree-BLAST. Based on synthesis report, the design uses 55% of the slices. Our design has longer array size and consumes less resource, comparing with Tree-BLAST built on XC4VLX160 with 78% of the slices. For synthesis performance comparison, we also implement our architecture with 2048 components on FPGA XC2VP70 and FPGA XC4VLX160. Results are shown in Table 1. 

We constructed two systolic arrays with 1024 and 3072 processing units to make a comparison in synthesis performance with the families of FPGA-based accelerators. Results are shown in Table 2.

#### 5.1.2. Storage Requirement

Systolic array-based architecture requires less storage than many WPRBS architectures. The storage consumption of our architecture mainly consists of sequence memory and hits FIFO. The memory usage is 3408 Kbits in total which is 20.3% of the memory capacity in XC5VLX110T. Since many WPRBS architectures need to record the position of each word in query sequence, they generally consume much more storage. For instance, RC-BLAST spends 64 K X 64bits memory to store the query index in Xilinx 4085XLA. Mercury and Mitrion have external SRAM to store the hash table because internal memory is not enough for the record. The slow external memory access has negative impact on the algorithm implementation efficiency. Compared to these methods, our architecture saves storage, avoids the complexity of memory access, and simplifies the FPGA layout and routing.

#### 5.1.3. Word Scanning Speed

Most WPRBS architectures could search at most one word per clock cycle. The word scanning speed in Mercury BLASTn is 96 M matches/s. The speed of Multiengine BLASTn Accelerator which applies 64 identical parallel engines reaches 6400 M matches/s. The word scanning speed of our Multiple Hits Detection Module approach achieves 14450 M matches/s which is over 2 times than related work. 

### 5.2. Comparing Execution Time to Software Versions

Our design is mapped to the Xilinx XC5VLX110T FPGA to accelerate the first two steps of NCBI BLAST family programs. Query and subject sequences for experiments of different algorithms come from different databases. The situation is shown in Table 3.

As Table 3 illustrated, Swiss-prot from EBI [29] and Drosoph.net from NCBI BLAST are two databases in experiments. Swiss-prot is a protein sequence database while Drosoph.net is a database for DNA sequences. The Swiss-prot in our experiments is UniProtKB/Swiss-Prot protein knowledgebase release 2012_11. BLASTp is used for searching protein sequence against protein database. BLASTx is used for searching DNA sequence against protein database. Queries which are picked from Drosoph.nt translated into six-frame protein sequence before searching against Swiss-Prot. TBLASTn is used for searching protein sequence against DNA database. TBLASTx is sued for searching six-frame translation of DNA sequence against six-frame translation of DNA database. For each algorithm, we built 6 arrays with different sizes (128-3k) to carry different queries with different lengths. For each length, 15 queries with the same length are picked up randomly from the relevant database. Every query's running time was averaged over 3 identical runs. The final execution time for a certain array is the average of running time of 15 queries.  Table 4 shows the algorithms execution time of our architecture and software versions.

The execution time of the software versions in this table comes from the previous work [28]. The hardware execution time (HT) is less than software execution time (ST) for all cases. BLASTp software version only spends 1901 ms in searching database with a 128 long query while 25132 ms is taken when it is searching database with a 3-K long query. This is because a larger index is necessary when the sequence is longer. Index construction not only needs more time but also increases the size of searching object. In our architecture, however, the execution time does not increase so rapidly. This is because the searching time is equal to the sum of the time of database stream flowing through the array and the extension time of valid hits. The time of database stream flowing is determined by the size of database; the extension time of valid hits is influenced by the architecture design. In this experiment, the size of the database is not changed. The Hits Combination Block and the parallel design reduce the number of valid hits and their extension time. The architecture demonstrates more speedup with longer sequences. For the same reason, the execution time of TBLASTn, BLASTx, and TBLASTx which are implemented on the FPGA does not increase sharply.

**Figure 13:**
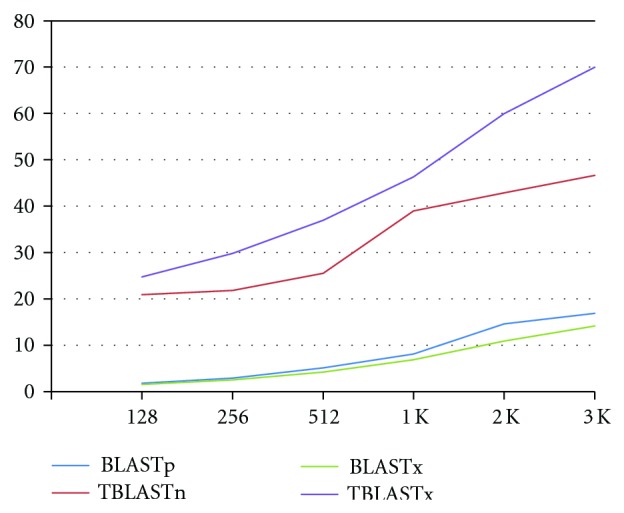
Speedup of the parallel FPGA architecture.

From Table 3, it can be seen that TBLASTn software version is slower than BLASTp software version for the same query length. The reason is that all DNA sequences are translated into the 6 possible potential proteins in TBLASTn software version before the searching. The translation time is not counted for the hardware version in our experiments because the translation is a one-time job, and results can be used for many other applications. For TBLASTx software version, both queries and the database are translated into the six possible potential proteins before searching. The translation time is not counted for the same reason as in TBLASTn. The speedup is shown in Figure 13. 

PatternHunter [30] which is implemented in Java applies a novel seed model called “space seeds” to accelerate BLAST algorithm. This software can run on desktops. It is faster and more sensitive than standard BLAST at that time. Our architecture could implement 3 steps of BLAST algorithm simultaneously because the FPGA is a good platform to parallel the BLAST algorithm. Paralleling BLAST algorithm on the FPGA improves the word scan speed a lot. PatternHunter has 48 M/4s word scan speed at most while ours could get 14450 M matches/s. 

## 6. Conclusion and Future Work

In this paper, we presented a systolic array-based FPGA parallel architecture for BLAST Algorithm. The architecture contains four different kinds of blocks which are Multiple Hits Detection Module, Hits Combination Block, Ungapped Extension Block, and Gapped Extension Block. Finding hits is the most computationally time-consuming step in BLAST. We designed the Multiple Hits Detection Module to improve the efficiency of the bottleneck step. The Multiple Hits Detection Module based on the systolic array could find more than one hit in only one clock cycle. The performance is faster than WPRBS searching approach used in other proposed architectures cited in this paper. In order to speedup hits searching, Hits Combination Block between Multiple Hits Detection Module and Ungapped Extension Block merges adjacent overlapping hits into one hit in order to speedup the extension step. Lastly, we proposed a parallel architecture for Ungapped Extension Block as Figure 12 illustrated. We have implemented Multiple Hits Detection Module, Hits Combination Block as well as Ungapped Extension Block. Based on the ISE synthesis report, we compared our architecture performance to the performance of other related work. The results showed advantages of our architecture in four aspects. First, compared to Tree-BLAST, a twice longer array can be built in our architecture with less FPGA resource. Secondly, our architecture only needs less memory space. Thirdly, the word scanning speed of our architecture faster than Mercury BLASTn's word scanning speed. Last, compared to BLASTp software, our architecture is more suitable for dealing with longer sequences. In the future, we hope to design an efficient architecture for Gapped Extension Block. To satisfy more users' requirement, we also want to modify our design to satisfy nucleotide searches. The Multiple Hits Detection Module needs to be changed, for instance, 3-input AND gate should be changed to 11-input AND gate. The clock_division block needs to be redesigned to guarantee the pipeline works correctly, more details will be addressed in the further design. Our architecture is only available for users who use Xilinx XC5VLX110T FPGA because we only test it on that FPGA to guarantee the correctness of its behavior and timing. After enough tests on different devices, the design would be available for more users who apply different FPGAs. 

## Figures and Tables

**Pseudocode 1:**
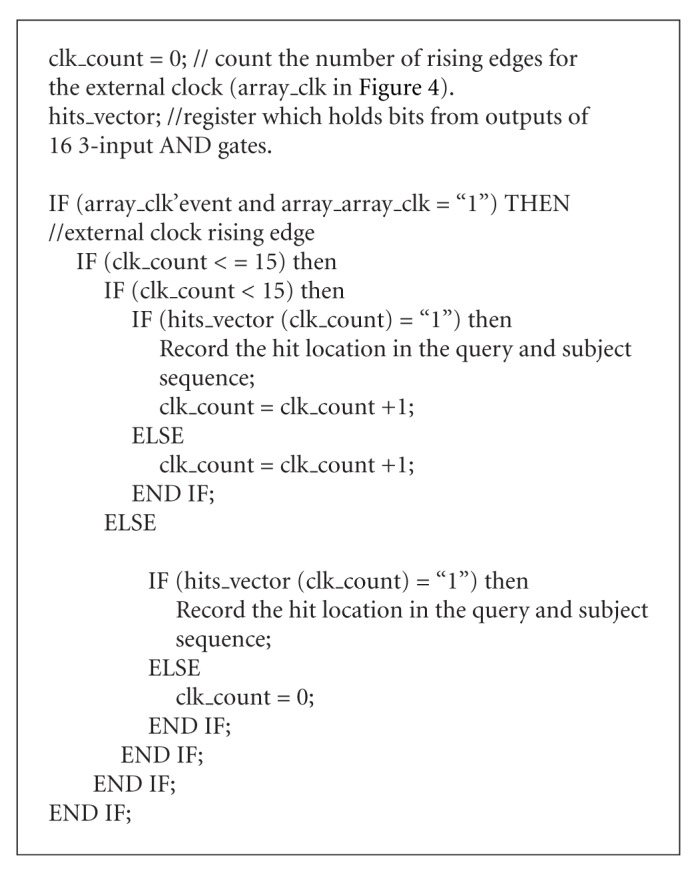


**Table 1:** Synthesis performance comparison between tree-BLAST and ours.

	Our architecture			Tree-BLAST	
FPGA	XC5VLX110T	XC2VP70	XC4VLX160	XC2VP70	XC4VLX160
Array size	2048	2048	2048	600	1024
Slice (%)	55	87	59.2	—	78
Memory (%)	20.3	42	28	—	88
Clock (MHz)	136	145	158	110	178

**Table 2:** Synthesis performance comparison between families of FPGA-based accelerators and ours.

	Our architecture			Families of FPGA-based accelerators	
FPGA	XC5VLX110T	XC2VP70	XC4VLX160	XC2VP70	XC4VLX160
Array size	2048	1024	3072	1024	3072
Slice (%)	55	58	69	60	71
Memory (%)	20.3	10.1	33.5	11	38
Clock (MHz)	136	125	166	140	189

**Table 3:** Experimental database for different algorithms.

Software version	Experiments data source	Experiments data size
BLASTp/BLASTx	Swiss-Prot	532146 sequences, 188719038 characters
TBLASTn/TBLASTx	Drosoph.nt	1170 sequences, 122655632 characters

**Table 4:** Execution time of our architecture and software versions.

Query length	BLASTp		TBLASTn		BLASTx		TBLASTx	
	ST(ms)	HT(ms)	ST(ms)	HT(ms)	ST(ms)	HT(ms)	ST(ms)	HT(ms)
128	1901	1050	3203	153	1594	1030	4031	163
256	3087	1058	3641	167	2641	1042	5906	198
512	5603	1092	5156	202	4378	1074	9978	270
1 K	9327	1150	9891	254	7828	1135	16266	351
2 K	17814	1220	15438	360	13187	1210	27703	462
3 K	25132	1490	20328	436	18875	1331	37500	536
